# Preliminary In Vitro Assessment of Decellularized Porcine Descending Aorta for Clinical Purposes

**DOI:** 10.3390/jfb14030141

**Published:** 2023-03-02

**Authors:** Martina Casarin, Tiago Moderno Fortunato, Saima Jalil Imran, Martina Todesco, Deborah Sandrin, Massimo Marchesan, Gino Gerosa, Filippo Romanato, Andrea Bagno, Fabrizio Dal Moro, Alessandro Morlacco

**Affiliations:** 1 Department of Surgery, Oncology and Gastroenterology, Giustiniani 2, 35128 Padua, Italy; 2 L.i.f.e.L.a.b. Program, Consorzio per la Ricerca Sanitaria (CORIS), Veneto Region, via N. Giustiniani 2, 35128 Padua, Italy; 3 Department of Cardiac, Thoracic Vascular Sciences and Public Health, University of Padua, via Giustiniani 2, 35128 Padua, Italy; 4 Department of Industrial Engineering, University of Padua, via Marzolo 9, 35131 Padua, Italy; 5 Department of Physics and Astronomy ‘G. Galilei’, University of Padova, via Marzolo 8, 35131 Padua, Italy; 6 Consultant of Animal Welfare and Food Inspection, 35100 Padua, Italy; 7 Laboratory of Optics and Bioimaging, Institute of Pediatric Research Città della Speranza, 35127 Padua, Italy

**Keywords:** descending aorta, decellularization, detergents’ permeabilization, regenerative medicine, tissue–engineering, biomaterial, conduits reconstruction

## Abstract

Conduit substitutes are increasingly in demand for cardiovascular and urological applications. In cases of bladder cancer, radical cystectomy is the preferred technique: after removing the bladder, a urinary diversion has to be created using autologous bowel, but several complications are associated with intestinal resection. Thus, alternative urinary substitutes are required to avoid autologous intestinal use, preventing complications and facilitating surgical procedures. In the present paper, we are proposing the exploitation of the decellularized porcine descending aorta as a novel and original conduit substitute. After being decellularized with the use of two alternative detergents (Tergitol and Ecosurf) and sterilized, the porcine descending aorta has been investigated to assess its permeability to detergents through methylene blue dye penetration analysis and to study its composition and structure by means of histomorphometric analyses, including DNA quantification, histology, two-photon microscopy, and hydroxyproline quantification. Biomechanical tests and cytocompatibility assays with human mesenchymal stem cells have been also performed. The results obtained demonstrated that the decellularized porcine descending aorta preserves its major features to be further evaluated as a candidate material for urological applications, even though further studies have to be carried out to demonstrate its suitability for the specific application, by performing in vivo tests in the animal model.

## 1. Introduction

The need for conduit substitutes is becoming more and more prominent in clinical practice. This is of avail not only for cardiovascular applications, e.g., the substitution of great vessels such as the aorta, but also for other tissues and organs, such as autologous intestine, which is used as urinary diversion. In case of cancerous urinary bladder, it has to be removed when conservative treatments (i.e., chemo- and radiotherapies) are unsuccessful. This surgical procedure is named radical cystectomy. Bladder cancer is one of the most common malignancies in the world: it is in the 10th place among malignancies and at similar level as a cause of mortality [1]. Bladder cancer incidence is sixth highest in the European Union, with age-standardized incidence rates of 19.1 for males and 4.0 for women [2,3]. The morbidity of radical cystectomy is significant as it is associated with 80% of problems, such a higher risk of malignant transformation, electrolyte imbalances, and mucus production [4].

In order to spare autologous tissues, several approaches for the creation of conduit substitutes have been tested already, including: synthetic polymers (e.g., polyglycolic acid, polylactic acid a polylactic-glycolic acid [5,6,7,8,9]), biological materials (e.g., collagen [10,11,12,13], hyaluronic acid, silk [14,15] and alginate [16]), acellular allogeneic and xenogeneic tissues (e.g., SIS [17,18,19,20], and decellularized bladder matrix [21,22]). However, the ideal substitute has not yet been identified [1,2]. Synthetic materials showed advantageous and dependable mechanical properties, but they still only have a modest degree of biocompatibility, thus evoking foreign body reactions. Conversely, naturally derived polymers are biocompatible and biodegradable, favouring tissue regeneration. Acellular tissue scaffolds, both allogeneic and xenogeneic, which were shown to maintain tissue microstructure, are rich in extracellular matrix (ECM) proteins such as collagen, elastin, and laminin, and growth factors, which provide the three-dimensional environment for growing cells, thus supporting tissue regeneration. The presence of ECM proteins and growth factors fosters tissue reconstruction and regeneration following in vivo implantation, giving host cells specific signals to migrate, grow, and proliferate into the scaffold. However, the main challenge regarding the use of acellular tissues is due to the development of an effective decellularization procedure, which can guarantee the proper elimination of cells and DNA residues to prevent immune rejection, while optimally preserving tissue’s ECM to promote recellularization and, eventually, tissue regeneration [23,24].

We recently published a study to propose small intestinal submucosa (SIS) [25] as a possible candidate material to create a tissue-engineered urinary conduit, thus sparing healthy bowel tissue. Despite its promising in vitro cytocompatibility, the main limitation is represented by tissue permeability, which can be overcome by a hybrid membrane: it is obtained by coupling an acellular biological tissue and a synthetic polymer [26]. A different solution can be represented by the exploitation of a thicker tissue with a tubular shape, such as the descending aorta. Therefore, the present study aims at evaluating the porcine descending aorta as an alternative tissue-engineered conduit, which can be used for several surgical applications such as aorta substitution or, in the specific area of urological surgery, for the resection of urinary diversions in the place of autologous intestine.

After the declaration of Triton X-100 transformation into an element having endocrine-disrupting characteristics [27], its use was restricted by the European Chemical Agency (ECHA), making the search for alternative decellularizing detergents necessary. Following a preliminary study of detergent’s permeabilization using methylene blue dye, two different decellularization procedures were exploited and their effectiveness was compared by the standard analytical tools (e.g., histology, immunofluorescence, mechanical tests) in order to select the most effective one. Therefore, the decellularized descending aorta was further characterized in terms of sterility and cytocompatibility. The results obtained indicate that the porcine descending aorta can been advantageously considered as a candidate material for the construction of tubular conduits for several surgical applications.

## 2. Materials and Methods

### 2.1. Porcine Descending Aorta Procurement

Porcine descending aortas were obtained from an abattoir and treated within 3 h of the animals’ sacrifice. The protocols followed by the abattoir (certified ISO 9001, ISO 22000:2005, ISO 22005:2008) were consistent with EC regulations 1099/2009 concerning animal health and protection at the moment of slaughter, under the direction of the Italian Ministry of Health (Food and Consumer Product Safety Authority). External fibrous and adipose tissues were removed, paying special attention to prevent cutting collateral branches in order to simplify surgical sutures after the decellularization treatment. Conduits of 15 cm in length were obtained and washed with phosphate-buffered saline (PBS) until the solution became clear. Descending aortas were then split into two groups. In group A, tissues were directly treated for decellularization (decellularized procedure 1, D1), while in group B tissues were first grossly dried and stored at –80 °C and then subjected to the decellularization process (decellularized procedure 2, D2).

### 2.2. Descending Aorta Decellularization

Two different decellularization procedures were exploited and compared; they were implemented by optimizing an already established protocol previously proposed for heart valves (TriCol) with the use of Triton X-100 [28,29] and a recently published protocol for porcine aortic valves where Tergitol was used instead of Triton X-100 [30].

#### 2.2.1. Decellularization Procedure 1 (D1)

Fresh descending aortas (group A) were treated under constant agitation, with protease inhibitors alternated with hypotonic and hypertonic solutions. Tissues were incubated with Tergitol (cat. no. 15S9, Sigma-Aldrich, Saint Louis, MO, USA) and sodium cholate (cat. no. C1254, Sigma-Aldrich) with intermediate washing in PBS or physiological solution, and subsequentially with an alcohol-based solution, followed by the endonuclease treatment (Benzonase^®^, cat. no. E1014, Sigma-Aldrich) to remove residual DNA fragments.

#### 2.2.2. Decellularization Procedure 2 (D2)

Fresh descending aortas were gently dried, frozen, and stored at –80 °C (group B). Afterward, native frozen aortas were thawed and then treated with procedure D1 modified with the addition of peracetic acid (PAA) to the final alcohol-based solution. Endonuclease (benzonase, cat. no. E1014, Sigma-Aldrich) was finally added to remove residual DNA fragments.

#### 2.2.3. Decellularization Procedure 3 (D3)

Fresh descending aortas (group C) were treated with the procedure D1 replacing Tergitol detergent with Ecosurf (cat.no A9779,0500, PanReac. AppliChem, Barcellona, Spain) at the same concentration. Procedure D3 was stopped before the step with sodium cholate, considering the different permeabilization capacity of the detergents as discussed in Section 3.1 (also Supplementary Materials).

After being decellularized with both procedures, acellular tissues were treated with antibiotics/antimycotics plus peracetic acid solution for sterilization.

### 2.3. Comparison of Permeabilization Capacity of Different Detergents

As a method of assessment of the effectiveness of the detergents used for decellularization under protocol D1 and D3, a comparison of permeabilization was performed using methylene blue dye staining, as suggested by Zhao et al. [31]. As the most critical steps of our decellularization procedure are the first three ones (i.e., inhibition of proteases, osmotic shock, and detergent treatment), we decided to perform the permeabilization analysis at the end of the third step. Consequently, circular sections of proximal (“prox”) and distal (“dist”) regions of porcine descending aortas (5 cm long) were divided into two groups (n = 3 for each group) and subjected to steps 1–3 of the decellularization procedures D1 (group Terg) and D3 (group Eco) to compare detergents’ permeabilization capacity (Ecosurf data are reported in Supplementary Section). After washing with PBS, samples were incubated with 0.05% methylene blue dye (Sigma, cat. no. M9140) at 37 °C overnight. Following rewashing in PBS, samples were cross sectioned in order to evaluate dye penetration throughout the tissue; consequently, the permeabilization efficacy of the detergents was assessed. Cross-section pictures were acquired using a Nikon D800 camera (objective 105f, 2.8 mm micro ED), segmented with Adobe Photoshop CC2019 and analysed by Matlab^®^ (Mathworks, Natick, MA, USA) to create a profile plot along the centre of the sliced samples by calculating histograms of segmented pictures and grey level plots along the samples’ thickness.

### 2.4. DNA Quantification

Native and decellularized samples were lyophilized, and the total DNA content was collected (5–10 mg) with D1 and D2 (n = 6 for each group), using a DNeasy Blood and Tissue Kit (cat. no. 69506, Qiagen, Valencia, CA, USA). Concentration was measured at 260 nm with a NanoDrop One Spectrophotometer (Thermo Scientific, Waltham, MA, USA) and with Qubit 2.0 (Thermo Fisher Scientific, Waltham, MA, USA) using a Qubit^TM^ 1X dsDNA HS Assay Kit (cat. no. Q33231, Thermo Fisher Scientific, Waltham, MA, USA). Total DNA values were divided by the dry weight.

### 2.5. Histological Stains

Cryosectioned native and D1/D2 tissue slices (6 µm thick) were processed for the histological analyses with a haematoxylin and eosin kit (cat. no. 04-061010, BioOptica, Milan, Italy) and Masson’s trichrome (cat. no. 04-010802, BioOptica, Milan, Italy) to visualize nuclei (in black), collagen fibres (in blue), and cytoplasm and muscle fibres (in red) and with an Alcian blue stain kit (pH 2.5, Mucin Stain, cat. no. ab150662, Abcam, Cambridge, UK) for the examination of hyaluronic acid and sialomucins. Supplier’s instructions were followed for each staining. Pictures were collected with an EVOS XL Core Cell Imaging System (Thermo Fisher Scientific, Waltham, MA, USA).

### 2.6. Immunofluorescence

Immunofluorescence was used to analyse the ECM and determine whether or not nuclei were present in the cryosectioned samples of descending aorta (6 µm thick) following the protocol described in [25]. Slides were incubated overnight at 4 °C with antibodies against collagen I (1:100, cat. no. C2456, Sigma-Aldrich, Saint Louis, MO, USA), collagen IV (1:200, cat. no. ab6586, Abcam, Cambridge, UK), and laminin (1:200, cat. no. L9393, Sigma-Aldrich, Saint Louis, MO, USA). For secondary incubation, Alexa Fluor 555 goat anti-mouse IgG (1:300, cat. no. A21422, Thermo Fisher Scientific, Waltham, MA, USA) and goat anti-rabbit IgG (1:300, cat. no. A27039, Thermo Fisher Scientific, Waltham, MA, USA) secondary antibodies were incubated for 1.5 hrs. at RT. Finally, DAPI (NucBlue Fixed Cell Strain ReadyProbes reagent, cat. no. R37606, Thermo Fisher Scientific, Waltham, MA, USA) was used to counterstain nuclei.

An autofluorescence signal (excitation wavelength of 480–440 nm, emission wavelength of 527–530 nm) was used to detect elastin on tissue samples.

Cross-sectional pictures were taken with an epifluorescence microscope (Leica AF6000) connected to a Leica DC300 digital camera and equipped with LAS AF Software (Leica Micro-Systems, Wetzlar, Germany). ImageJ software was used for post imaging analysis.

### 2.7. Two-Photon Microscopy

Native and decellularized specimens of descending aorta were analysed with a custom-made two-photon multimodal microscope [32] to evaluate the decellularization’s effects on collagen structure by measuring the second harmonic generation (SHG) signal [33]. The same parameters described in [25] were applied.

For numerical assessments, the RAW uncompressed pictures were analysed with ImageJ software [34]. SHG intensity was considered directly proportional to the content of collagen. Following this, collagen fibre distribution was evaluated through fast Fourier transform (FFT) and coherency (C) parameters, to determine the fibres’ local orientation (values close to 0 indicate isotropic regions, whereas values close to 1 indicate a highly oriented structure), using the plug-in OrientationJ [35,36].

### 2.8. Mechanical Tests

A Mitutoyo digital calliper (model ID-C112XB, Aurora, IL, USA) was used to measure sample thickness. Tests for uniaxial tensile loading were conducted using a specially designed apparatus (IRS, Padova, Italy). Dog-bone-shaped specimens (gauge length of 5 mm and width of 2 mm as described [25,37]) were cut following the ASTM D1708-13 standard regarding small-size tissues. The samples were kept hydrated with saline solution for the tests, which were conducted at room temperature. Samples (n = 9) were pre-loaded up to 0.1 N, then stretched to rupture at a rate of 0.2 mm/s. Data were evaluated with an in-house implemented Matlab^®^ script (Mathworks, Natick, MA, USA) in order to obtain the following parameters from the stress–strain curve of each sample: engineering stress (MPa) was computed by dividing the initial cross-sectional area of the sample by the tensile force detected by the loading cells and strain (%) was computed as the grip-displacement to gauge-length ratio. Ultimate tensile strength (UTS) and failure strain (FS) were then determined as the maximum resistance and maximum elongation that each sample achieved. Two Young’s moduli (E_1_ and E_2_) were considered as the slope of two linear portions in the stress–strain curve: E_1_ was calculated between 1–10%, while E_2_ between 140–150%. The E_1_ modulus characterizes the first phase of the stress–strain curve, where stresses are low and only elastic fibres are strained; the E_2_ modulus characterizes the second phase of the curve, where collagen fibres begin to bear the load [38,39].

Aorta samples were examined circumferentially and longitudinally. Data statistical analysis was performed using GraphPad Prism software version 7.00 for MacOS (San Diego, CA, USA).

### 2.9. FTIR Analysis

In order to evaluate if and how much decellularization affects tissue composition, Fourier transform infrared (FTIR) spectroscopy using a Nicolet iS-50 spectrometer (Thermo Fisher Scientific, Waltham, MA, USA) with an attenuated total reflectance (ATR) accessory was used to process samples from native (n = 3) and decellularized with D2 (n = 3) swine descending aortas. Details about the apparatus were reported in our previous work [25].

Before the analysis, samples were cut into squares of 10 × 10 mm^2^ and equilibrated for 3–4 h in deuterium oxide (Janssen, Beerse, Belgium) to reduce the interference of water bands in the amide-I region [40,41]. A Matlab^®^ script (Mathworks, Natick, MA, USA) was used to analyse the data [42].

### 2.10. Hydroxyproline Quantification

Hydroxyproline quantification on freeze-dried native (n = 3) and decellularized (n = 3) descending aorta samples with D2 (≈5 mg lyophilized dry tissue) was made with a hydroxyproline assay kit (cat. no. MAK008, Sigma-Aldrich, Saint Louis, MO, USA), respecting the guidelines provided by the manufacturer as previously reported [25]. Absorbance was measured at 560 nm with a Spark 10M microplate reader (Tecan, Mannedorf, Switzerland). A hydroxyproline standard solution given by the manufacturer was used to create standard curves. The amount of hydroxyproline was determined as µg hydroxyproline/mg dry tissue.

### 2.11. Descending Aorta Sterilization

D2 tissue samples were subjected to sterilization with a combination of antibiotics and antimycotics (AA) solution at 37 °C for 1 day followed by 3 h treatment with 0.1% *v*/*v* peracetic acid (PAA), as described elsewhere [43].

### 2.12. Sterility Assessment

Circular samples (8 mm diameter) of native, decellularized, and eventually sterilized tissues (n = 6 for each group) were exposed to two different sets of culture media accordingly to the standardized guidelines of the European pharmacopoeia [44] to qualitatively investigate sterility, as already performed in our previous study [25]. Thioglycolate medium (cat. no. T9032, Sigma-Aldrich, Saint Louis, MO, USA) incubated at 35 °C and soya-bean casein digest medium at RT (cat. no. 22092, Sigma-Aldrich, Saint Louis, MO, USA), were utilized to find aerobic/anaerobic bacteria and fungi over 14 days. In all three groups, turbidity was visually evaluated and images were taken periodically.

### 2.13. Tests for In Vitro Cytotoxicity

Contact assays were used to assess the in vitro cytotoxicity of decellularized with D2 samples in line with ISO 10993 part 5 on the biological evaluation of medical devices. [45]. Circular patches (8 mm diameter) were cut from decellularized and sterilized descending aorta and fit into 48-well plate in aseptic conditions to seed cells on the intima side.

Samples were first incubated with Mesenchymal Stem Cell Growth Medium 2 (cat. no. C-28009, PromoCell, Heidelberg, Germany) containing SupplementMix (cat. no. C-39809, PromoCell) and 1% penicillin-streptomycin at 37 °C. Following this, bone-marrow-derived human mesenchymal stem cells (cat. No. C-12974, PromoCell) were seeded at a density of 20,000 cells/cm^2^ on the samples and cultivated for 1, 7, and 14 days. Immunofluorescence, live/dead staining, a metabolic proliferation test, and dsDNA quantification were used to measure cell viability and proliferation.

#### 2.13.1. Live/Dead Assay

At day 1, day 7, and day 14 cell viability was assessed using the live/dead viability/cytotoxicity kit (cat. No. MP 03224, Thermo Fisher Scientific, Waltham, MA, USA) following the previously described procedure [25]. Epifluorescence pictures were acquired with an Olympus IX71 microscope.

#### 2.13.2. WST Assay

In order to assess cell viability and proliferation, a WST-1 Cell Proliferation and Cytotoxicity Assay kit (cat. No. AR1159, Boster, Pleasanton, CA, USA) was used at the end of each time point (sample number n = 4 for each time point), following the procedure described in [25]. Using a microplate reader (Spark 10M Tecan, Tecan, Männedorf, Switzerland), absorbance was measured at 450 nm. The fluorescence levels of each experimental well were averaged, and the fluorescence values of the no-cell control wells were subtracted.

#### 2.13.3. Whole Mount Immunofluorescence

Whole mount immunofluorescence was carried out following the same protocol described in [25] using phalloidin-Atto 647N (1:200, cat. No. 65906, Sigma-Aldrich) and DAPI to stain F-actin filaments and nuclei on seeded patches at day 1, day 7, and day 14.

Z-stack images from the intimal side were taken using an epifluorescence microscope (Leica AF6000) connected to a Leica DC300 digital camera and equipped with LAS AF Software (Leica Micro-Systems, Wetzlar, Germany). ImageJ software was used for post imaging analysis.

## 3. Results

### 3.1. Evaluation of Detergents’ Permeabilization

**Figure 1:**
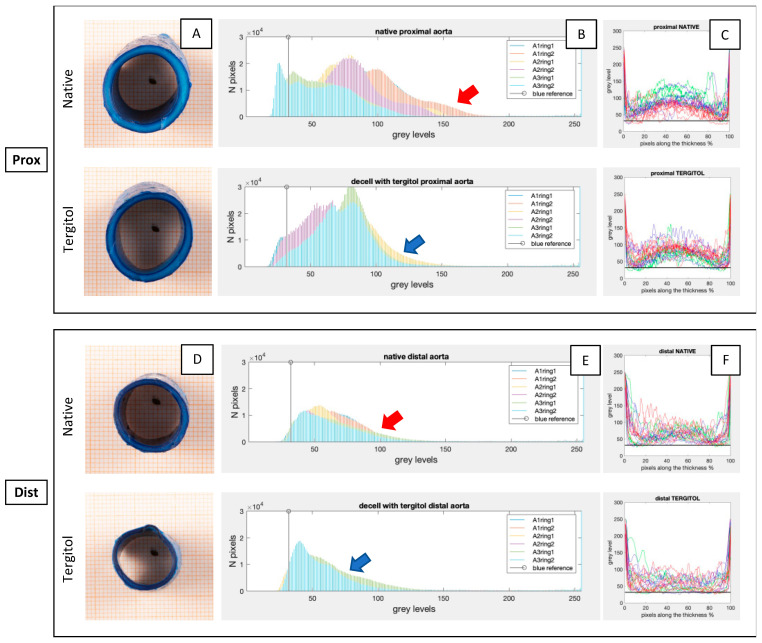
Penetration assessment of methylene blue dye in native and Tergitol-treated samples at the end of the third step of the decellularization procedure. Circular samples of descending aortas in proximal (**A**) and distal (**D**) regions were stained with methylene blue dye and cut in the middle to acquire pictures of the cross-sectional area. Pictures were then segmented and analysed to obtain the histograms of the occurrence of each grey level (**B**,**E**) and then the graphs of grey levels along the thickness (%) of samples (**C**,**F**). Vertical black lines in (**B**,**E**) and horizontal black lines in (**C**,**F**) represent the reference grey level of methylene blue dye. Red arrows highlight wider curves in higher grey level values in native groups, while blue arrows show narrowed curves with higher peaks for the Tergitol-treated samples. Greyscale: 0 = black, 255 = white.

To preliminarily assess the permeabilization potential of the detergents used for decellularizing the descending aortas (i.e., Tergitol in Figure 1 and Ecosurf in Figure S1), the diffusion of methylene blue dye through the vascular wall was quantified and it was taken as an indirect estimation of detergent penetration. Pictures of cross-sectional areas from descending aorta samples are reported in Figure 1A,D. Since the number of lamellae layers increases with the thickness of the arterial wall, which is proportional to the diameter, we decided to consider separately the proximal and distal regions in order to further characterize descending aorta. Histograms of segmented images (Figure 1B,E) of both proximal and distal regions show the occurrence of pixel numbers for each grey level, revealing lower and broader curves in the case of native tissue (Figure 1B,E, red arrows), while Tergitol gives higher evidence of a sharp peak followed by a flattened curve (Figure 1B,E, blue arrows). Moreover, a greater variability was found in the case of native tissues when compared to Tergitol-treated samples. Generally, the proximal regions presented wider curves than the distal ones due to the higher thickness of the tissue, suggesting an augmented penetration of methylene blue dye in the distal region because of its thinness. In order to quantify the penetration of methylene blue dye, grey levels along the thickness of the samples are reported (Figure 1C,F): 0% represents the outer layer of the aorta (adventitia), 50% represents the central part (media), and 100% represents the inner layer (intima). The increased degree of permeabilization along sample thickness is highlighted by the flattening of the curve in the middle: in the case of native tissue, higher grey levels at around 50% thickness indicate a lower penetration of methylene blue dye, while in the case of Tergitol-treated samples lower values were found.

### 3.2. Evaluation of Decellularization Efficacy

**Figure 2:**
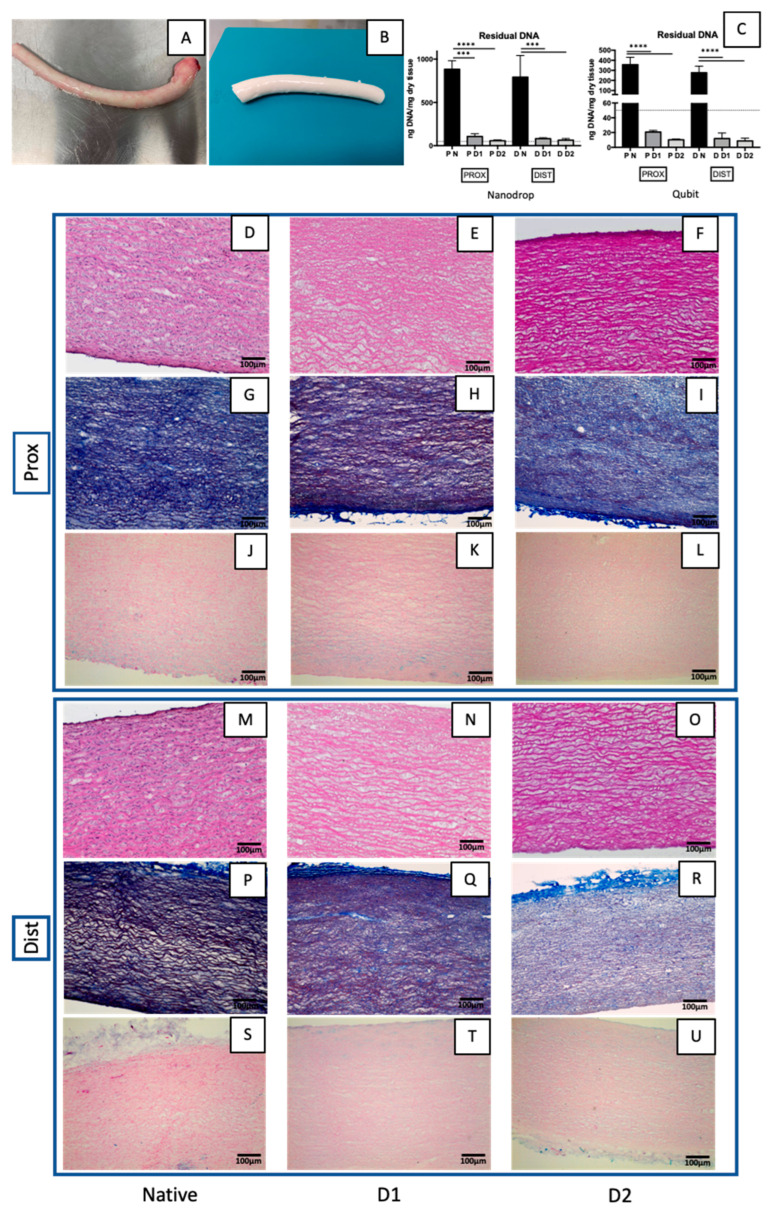
Descending aorta before (**A**) and after decellularization (**B**). DNA quantification (**C**) made in native and decellularized descending aortas using D1 and D2: with NanoDrop on the left and with Qubit on the right. Data on graphs display the mean ± SD. Data were analysed by *t*-test. **** *p* < 0.0001 and *** *p* < 0.001. Histological analyses of native and decellularized descending aortas with D1 and D2: haematoxylin and eosin staining illustrates the successful nuclei elimination in decellularized tissue (**D**–**F**,**M**–**O**). Collagen and elastic fibres are revealed by Masson’s trichrome (**G**–**I**,**P**–**R**). Alcian blue shows the presence of glycoproteins (**J**–**L**,**S**–**U**).

After decellularization with Tergitol, descending aortas macroscopically appeared well preserved and whiter than the native tissue because of the removal of cells and blood residues (Figure 2A,B).

The DNA amount in native and decellularized samples was measured using two different techniques: the first method measures the UV absorbance at 260 nm (Nanodrop), and the second one measures the intensity of a fluorescent dye that specifically binds to double-stranded DNA (Qubit). Decellularized tissue (n = 3) has significantly less DNA than native tissue (n = 3), according to DNA quantification (Figure 2C). The DNA content in the decellularized descending aortas (D1 and D2) was under the threshold of 50 ng dsDNA/mg dry tissue, which is one of the criteria for successful decellularization stated by Crapo et al. [46].

The descending aorta is a smooth great vessel that narrows in the proximal to distal direction. Microscopically, histological analysis (haematoxylin and eosin and Masson’s trichrome staining) confirmed the conservation of fibres after both Tergitol-based decellularization processes, while all the nuclei were removed both in the proximal (Figure 2D–L) and distal (Figure 2M–U) regions.

### 3.3. Structural and Biomechanical Characterization

**Figure 3:**
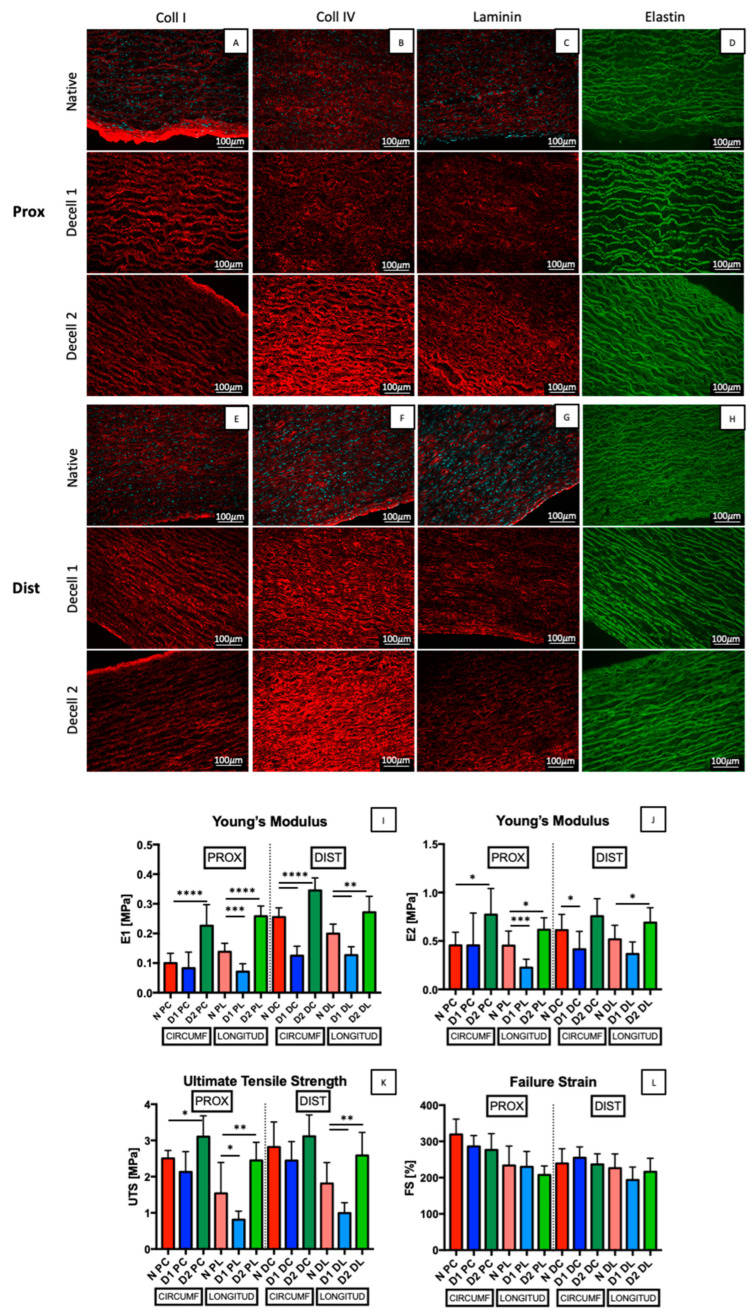
(**A**–**J**): Immunofluorescence of collagen I (**A**,**E**), collagen IV (**B**,**F**), laminin (**C**,**G**) in red, elastin (**D**,**H**) in green and DAPI in cyan. Scale bar: 100 µm. Young’s moduli (E1 and E2), ultimate tensile strength (UTS), and failure strain (FS) are stated correspondingly in (**I**–**L**) comparing native and descending aortas decellularized with D1 and D2 in proximal and distal regions, along longitudinal and circumferential directions. Data on graphs display mean ± SD. Data analysed by *t*-test. **** *p* < 0.0001, *** *p* < 0.001, ** *p* < 0.01 and * *p* < 0.05 (n = 9).

Collagen I and IV fibres were well-maintained (Figure 3A,B,E,F) in both D1 and D2 groups in comparison with the native tissue, showing a denser distribution nearby the adventitia in the case of collagen I. Collagen IV presented an increased signal in case of D2, especially in the proximal region, since fibres became more compacted after decellularization. Laminin (Figure 3C,G) did not seem to be affected by the decellularization processes either. Comparing the proximal and the distal regions, the latter appeared more compacted in all groups (native, D1 and D2). The content of elastin (Figure 3D,H), which is mainly present in tunica media showing parallel elastic fibres, was preserved in both decellularized groups.

Uniaxial mechanical tests were performed in order to get an estimation of the descending aorta’s mechanical behaviour. A stiffness increase was detected both circumferentially and longitudinally after D1 and D2. In order to evaluate it more precisely, the Young’s modulus was calculated in the first (E_1_, 1–10%) and in the last (E_2_, 140–150%) phases of deformation. Thus, the E_1_ values revealed (Figure 3I) a decrease in stiffness along the longitudinal direction of the proximal region and a decrease in both directions in the case of the distal region, for D1 samples; a stiffening rise was found in both directions and in both regions for D2 samples. Similarly, in the case of E_2_ (Figure 3J), a decrease was detected along the longitudinal direction of the proximal region and in the circumferential direction of the distal region for D1 samples, while an increase along both directions was found in proximal region but only along longitudinal direction of the distal one for D2 samples. Correspondingly, a similar trend was observed for UTS values (Figure 3K), which decreased in D1 samples, but with statistical differences only in the longitudinal direction of both regions. In the case of D2 samples, an increasing trend in UTS values was observed in both directions and both regions: they were statistically different in both directions of the proximal region and in the longitudinal direction of the distal one. This variation was not found in FS values (Figure 3L), suggesting that the decellularization processes might have changed the stiffness and elasticity of the tissue, but not how long it would last before failing.

### 3.4. Collagen Evaluation with Two-Photon Microscopy

**Figure 4:**
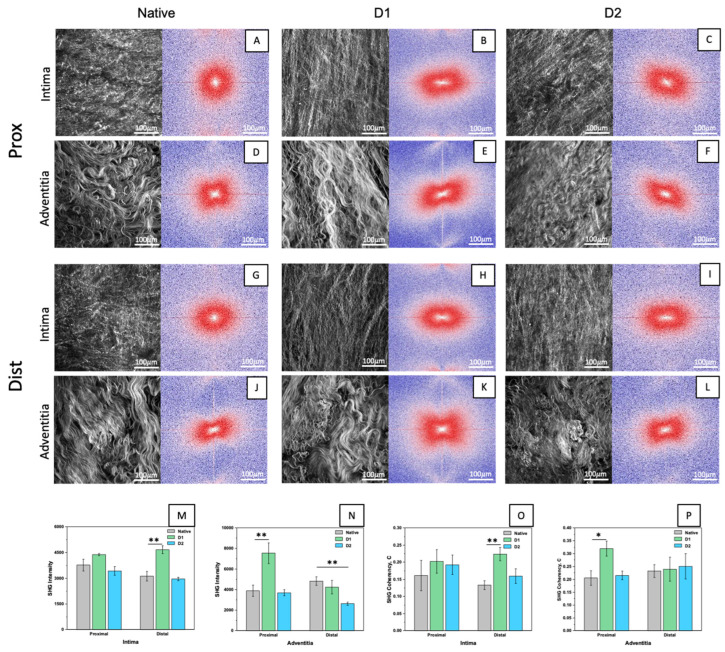
Z–stack of max intensity of phase contrast of native and decellularized descending aortas with D1 and D2 in proximal and distal regions and demonstrative FFT (**A**–**L**). SHG intensity values from z-stack images in intima (**M**) and in adventitia (**N**). Coherency analysis from z-stack in intima (**O**) and in adventitia (**P**). Data on graphs show mean ± SD. Data analysed by Dunnett’s multiple comparisons test (native as control group). ** *p* < 0.01, * *p* < 0.05.

Collagen fibres of native and decellularized (D1 and D2) descending aortas were analysed by two-photon microscopy (Figure 4A–L). The quantity of collagen was proportional to SHG intensity (Figure 4M,N), while local fibre orientation was assessed with the coherency € (Figure 4O,P) and FFT (Figure 4A–L). SHG intensity significatively increased in D1 samples, since collagen fibres are distributed more densely, in the intima of the distal and in the adventitia of the proximal region. In the case of D2, a significant difference was detected only for adventitia in the distal region. The analysis of the coherency parameter highlighted significant changes only between native and D1 samples in the intima of the distal and in the adventitia of the proximal region. No significant differences were found between native and D2 samples. These results were confirmed by the FFT images.

In the light of these results (i.e., increased nuclei removal and ECM maintenance), the D2 protocol was preferred for descending aorta decellularization. Therefore, the D2 protocol was selected and used to treat further aorta samples to perform in-depth analyses with regard to ECM composition (i.e., hydroxyproline quantification and FTIR), sterility, and cytocompatibility.

**Figure 5:**
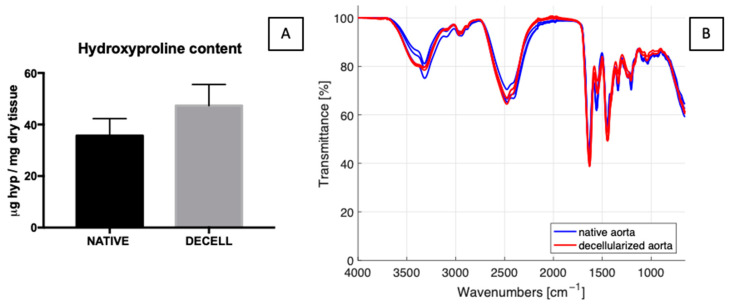
Hydroxyproline (**A**) quantification is reported: absence of significant difference was found between native and D2 decellularized tissues (data on graphs display mean ± SD). Data were analysed by *t*-test. FTIR spectra are reported (**B**), showing that the characteristic peaks are preserved.

Hydroxyproline quantification of native and D2 samples revealed no significant differences (Figure 5A).

The FTIR spectra of native and D2 decellularized samples overlapped; thus, comparing the characteristic peaks of both groups (Figure 5B), spectra largely coincide, and common peaks are detected at 1631 cm^−1^ (amide I and triple helix), 1556 cm^−1^ (amide II), 1446 cm^−1^ (every type of collagen), 1336 cm^−1^ (exclusive for collagen I and IV), 1205 cm^−1^ (exclusive for collagen IV and V), 1081 cm^−1^ (exclusive for collagen V and VI) and 1035 cm^−1^ (every type of collagen) [47,48,49].

### 3.5. Sterility Assessment and Cytocompatibility Test

In order to assess the sterility of decellularized aortas, turbidity tests were conducted in accordance with the European pharmacopoeia’s guidelines [44], using thioglycolate and soya-bean casein digest media broths. Prior to decontamination, native and D2 decellularized tissues displayed increased turbidity in all the tubes of both mediums. No difference in colour or turbidity was found in tissue samples that had been decellularized and sterilized (Figure S2A,B). Turbidity tests results are summarized in Table 1.

**Figure 6:**
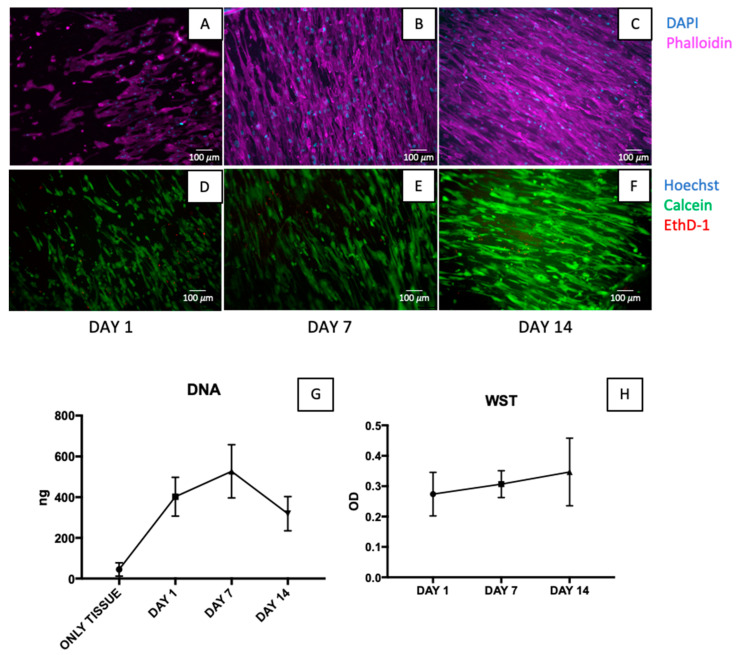
Cytocompatibility tests. Seeded samples were stained with phalloidin (magenta) and DAPI (cyan) at day 1 (**A**), day 7 (**B**), and day 14 (**C**). The number of cells gradually increased throughout the course of the experiment, and cells also organized themselves along collagen fibres (here z-stacks of epifluorescence images are stated). Live/dead staining on seeded descending aortas at day 1 (**D**), day 7 (**E**), and day 14 (**F**) are depicted (nuclei in blue, live cells in green and dead cells in red). There were not many dead cells found, but it was clear that there were more live cells with time. Scale bar: 100 µm. DNA quantity (**G**) and optical density (OD) (**H**) on tissue seeded are illustrated, showing no significant difference. Dunnett’s multiple comparisons test was executed (n = 3).

In order to perform cytocompatibility tests, decellularized samples were seeded on the intimal side with MSCs and then analysed at day 1, day 7, and day 14. Nuclei and cytoskeleton were counterstained with DAPI and fluorescently labelled phalloidin, respectively, demonstrating the re-organization of cells over time onto the intimal side (Figure 6A–C).

Live/dead staining was performed and the number of viable cells significantly increased from day 1 to day 14 according to calcein AM staining, while EthD–1 staining did not reveal dead cells in the same timeframe (Figure 6D–F).

DNA quantification of seeded patches was performed on day 1, day 7, and day 14, revealing an increasing trend from day 1 to day 7 and a decreasing trend up to day 14. There was no noticeable difference between day 1, day 7, and day 14 (Figure 6G).

The metabolic WST–1 assay was used to measure cell proliferation as well at days 1, 7, and 14, which showed no significant differences (Figure 6H).

## 4. Discussion

Currently, the fabrication of high calibre conduits is a major challenge for tissue engineering to replace tubular anatomical structures, from blood vessels to urological tissues. The radical cystectomy is the gold-standard treatment for urinary bladder cancer and implies the removal of bladder and the re-establishment of the urinary flow by creating a urinary diversion by means of an autologous intestinal portion. After surgery, the most frequent hurdles are associated with the removal of intestinal segment, which can result in the formation of adhesions, gastrointestinal (GI) tract bleeding, bowel outflow, the development of fistulas, and the progress of metabolic disorders depending on the intestinal portion utilized, and on the length and the type of diversion [4,50,51,52,53]. Therefore, these high-risk complications spur researchers to discover an effective alternative to autologous grafts.

A possible solution can be achieved thanks to a tissue-engineering approach, which has been recently proposed for tissue and organ substitution [23,24,54,55]. For the aforementioned urological application, the main requirements have been extensively described [55,56]; in particular, they regard the need for a watertight scaffold, which must maintain the conduit’s patency, compliance, and elasticity. The need for a material able to prevent urine leakage must be weighed against the requirement to permit sufficient cell motility, adhesion, proliferation, and differentiation.

At present, the descending aorta has never been considered for application as a urinary conduit. Indeed, there are very sparse and old studies regarding arterial and vein grafts used as ureteral substitutes, which were however not successful [57,58,59,60,61,62]. The aorta has been used for cardiovascular applications, and several decellularization procedures have been investigated, including the use of dimethyl sulfoxide as a penetration enhancer in order to reduce the exposure time to sodium dodecyl sulphate (SDS), supercritical carbon dioxide (sc–CO_2_) in order to significantly reduce treatments duration, besides SDS, trypsin, ethanol, supercritical dimethyl ether (DME), and sodium deoxycholate [63,64,65,66,67].

In the present study, the use of aggressive solutions was avoided by exploiting two milder detergents (Tergitol and Ecosurf) as alternatives to Triton X-100, which was recently declared toxic. We proposed the evaluation of their permeabilization efficiency using methylene blue dye staining, as suggested by Zhao et al. [28] in pig pancreas, in order to quickly select the most appropriate detergent. In addition to the graphs of grey level occurrence along the wall thickness, we proposed the analysis of the histograms of cross-sectional areas of circular descending aortas samples to analyse detergents’ permeabilization. We found an increased permeabilization by Tergitol and consequently an increased penetration of methylene blue, leading to narrower peaks in the histograms and, overall, closer values to the reference (methylene blue) in the graphs of grey levels along the thickness, both in proximal (thicker) and distal (thinner) regions. This result is in accordance with a recent study [31] in which Tergitol was successfully used to decellularize porcine aortic valve, while no studies have been reported in the case of Ecosurf-based decellularization.

Once the appropriate detergent was identified, we compared two optimized Tergitol-based decellularization procedures in order to avoid any adverse immune response [54,68], while simultaneously preserving ECM components. Four aspects are mandatory to guarantee an effective decellularization: cells and DNA complete removal, the preservation of proteins, and maintenance of mechanical features [69]. Thus, several analyses were performed, starting from DNA quantification, histological assays, hydroxyproline quantification, two-photon microscopy, and mechanical and cytocompatibility assays.

Both the exploited decellularization protocols (D1 with fresh tissue and D2 with a previously freeze–thaw step and an additional step with PAA) were effective in dsDNA removal, since its concentration was below the limit of 50 ng/mg in dry tissue [46]. Unlike other studies involving the use of SDS, which was associated with the disruption of protein–protein interactions, leading to structural deformation/alteration of ECM conformation and resulting in mechanically weak tissue [63], we demonstrated the maintenance of ECM main components such as laminin, elastin, and collagens (e.g., collagen I and IV): they are all prerequisites for cells to infiltrate the matrix [54], while effectively removing native nuclei. In detail, the descending aorta is an elastic artery, whose main components are elastin and collagen fibres, smooth muscle cells (removed by the decellularization processes), and a proteoglycan-rich ground substance. The components that give a concrete contribution to the mechanical properties are muscle fibres, elastin (in longitudinal direction), and collagen (which is randomly structured at low stresses and progressively stretches with increasing stresses). The initial elastic behaviour can be assigned to elastin fibres with a low Young’s modulus, while the final nonlinear behaviour with increasing deformations is due to collagen fibres. Consequently, elastin and smooth muscle fibres act at low deformations under physiological conditions, while collagen allows for the preservation of conduit wall integrity. Immunofluorescence confirmed the findings of other studies [70,71], which concluded that collagen fibrils in the media are significantly thinner than the adventitial ones.

Therefore, the stiffness of the aortic wall was found to be associated with the connective fibres, i.e., elastin and collagen [72]. Moreover, it was hypothesized [73] that an aorta’s stiffness depends on the fibre quantity rather than on their apparent density and that, for a same amount of fibres, their interaction increases when the wall thickness decreases. Even though it is well acknowledged that the aortic stiffness increases along the aortic tree from proximal to distal regions (elevated stiffness in the distal thoracic aorta is due to a larger contribution of collagen compared to proximal thoracic aorta) [74,75,76], in our study no significant change was observed between proximal and distal regions of both native and decellularized descending aortas at higher strains. This evidence suggests the suitability of the selected aortic segment for several surgical applications, including in the urological field, guaranteeing a constant mechanical behaviour along the conduit length after both decellularization procedures.

From the SHG images, we confirmed the findings of a previous study [74] regarding the higher density and corrugation of collagen fibres in the native proximal region compared to the distal one, where collagen fibres seem to be less crimped, with a more defined and traceable structure. When comparing native tissues to both decellularized groups (D1 and D2), we generally found significant differences in the case of D2-treated samples only in the adventitia of the distal region, while for D1 samples significant differences were found in SHG intensity of the intima of the distal and adventitia of the proximal region. These results (together with DNA quantification) suggest the greater suitability of the D2 protocol for decellularizing descending aortas. For this reason, we performed further investigations only on D2 samples. Both FTIR analysis and hydroxyproline quantification revealed no significant difference between native and decellularized samples, concluding that we were able to guarantee tissue decellularization whilst preserving collagen content.

After confirming the effective removal of cellular contents and ECM preservation after decellularization, it was crucial to assess the efficacy of the sterilization procedure. The ideal scaffold should favour interactions with cells, enhancing cell adhesion, growth, migration, and differentiation [77]. For this reason, a sterilization procedure that aims at getting rid of all forms of pathogens (bacteria, viruses, and yeasts) from the scaffold must not cause undesirable changes in physicochemical properties [78,79]. Usually, sterilization methods include chemical treatment (e.g., ethanol, ethylene oxide), antibiotics, irradiation (e.g., ultraviolet irradiation, gamma, and electron-beam irradiation) or heat, which have specific pros and cons. In the present case, the sterilization treatment was based on the use of a cocktail of antibiotics/antimycotics and peracetic acid, which was detailed in a previous study concerning decellularized SIS [25]. The use of antibiotics is a simple and effective way to inactivate bacteria by interfering with their essential processes, such as DNA replication and cell wall and protein synthesis. Antibiotics are also found to be effective against vegetative bacteria and spores [79]. PAA has a relative high penetration capacity that can efficiently inactivate several types of microorganisms, including vegetative bacteria, spores, enveloped and naked viruses, and fungi. As in the previous study [25], we were able to confirm the absence of contamination in treated samples, via turbidity tests accordingly to the pharmacopoeia protocol [44], demonstrating the efficacy of the sterilization procedure.

Before moving to the pre-clinical application in the animal model, it is crucial to evaluate the cytocompatibility of the scaffold in order to characterize its potentially harmful effects. The molecular events that can cause cellular, functional, and structural damage should be evaluated, since cells are extremely sensitive to leftover substances and can suddenly exhibit signs of toxicity in the presence of potentially hazardous compounds [80]. The cytocompatibility of a decellularized porcine descending aorta was assessed by direct contact assay in order to verify cell adherence to the scaffold, migration, and distribution. Human MSCs from bone marrow were chosen as the cell type to seed on tissue samples, since they can differentiate into numerous cells types, such as smooth muscle cells and endothelial cells [81,82,83], which would be present in both cardiovascular and urological applications. We found an increasing trend in the number of live cells and a clear arrangement along collagen fibres over time, with the almost total absence of dead cells. However, DNA data revealed an increasing trend from day 1 to day 7 but a decreasing one up to day 14. Correspondingly, the WST test showed an increasing metabolic activity over time, which was not statistically different. In our opinion, the last time point (day 14) is too long with respect to the static conditions in which cells were cultured. Therefore, we believe that a bioreactor must be used to assure proper (dynamic) cell-culturing conditions. Further studies will also focus on seeding cell types other than MSCs.

## 5. Conclusions

The need for replacement conduits is becoming more and more prominent, not only in the cardiovascular field for the creation of tissue-engineered grafts, but also for other clinical applications, such as in the urological field, i.e., the creation of a urinary diversion following radical cystectomy as an alternative to the autologous intestine. Porcine descending aorta has proven to be a promising candidate due to its shape, structure, and physicochemical and mechanical properties.

In the present study, two decellularization protocols have been compared: they were both able to guarantee effective DNA and cell removal, whilst assuring ECM preservation. The D2 protocol exhibited better performances and thus it was deeply investigated. The sterilization procedure was proven to be effective and safe prior to performing cytocompatibility tests by directly seeding MSCs on decellularized sterile tissue. Cells were shown to successfully attach and grow over time as demonstrated by time-course imaging of the seeded tissue. However, DNA quantification revealed an increasing trend only up to day 7. This limitation can be overcome using a bioreactor to optimize cell-culturing conditions. Future investigations will also exploit other tissue-specific cell types (e.g., endothelial and smooth muscle cells), taking into account the intended clinical translation of the impermeable conduit.

## Figures and Tables

**Table 1:** Results of turbidity tests on native, only decellularized, and decellularized + sterilized samples, compared with control group (only media).

Sample Name	Turbidity within 14 Days (Yes/No)
Native	Yes
Decellularized	Yes
Decellularized + antibiotic/antimycotic/PAA	No
Control (only media)	No

## Data Availability

Not applicable.

## Supplementary Materials

The following supporting information can be downloaded at: https://www.mdpi.com/article/10.3390/jfb14030141/s1, Figure S1: Penetration assessment of methylene blue dye in Ecosurf-treated samples at the end of the third step of the decellularization procedure in proximal and distal regions of descending aorta; Figure S2: Sterility assessment. Representative samples of turbidity tests of native, only decellularized and decellularized + sterilized samples are reported in comparison with only turbidity media (blank) both for thioglycolate medium (A) and soya broth medium (B) at day 14.

## References

[1] Sung H., Ferlay J., Siegel R.L., Laversanne M., Soerjomataram I., Jemal A., Bray F. (2021). Global cancer statistics 2020: GLOBOCAN estimates of incidence and mortality worldwide for 36 cancers in 185 countries. CA Cancer J. Clin..

[2] Serrano-Aroca Á., Vera-Donoso C.D., Moreno-Manzano V. (2018). Bioengineering approaches for bladder regeneration. Int. J. Mol. Sci..

[3] Alfred Witjes J., Lebret T., Compérat E.M., Cowan N.C., De Santis M., Bruins H.M., Hernández V., Espinós E.L., Dunn J., Rouanne M. (2017). Updated 2016 EAU Guidelines on Muscle-invasive and Metastatic Bladder Cancer. Eur. Urol..

[4] Bazargani S.T., Djaladat H., Ahmadi H., Miranda G., Cai J., Schuckman A.K., Daneshmand S. (2018). Gastrointestinal Complications Following Radical Cystectomy Using Enhanced Recovery Protocol. Eur. Urol. Focus.

[5] Atala A., Freeman M.R., Vacanti J.P., Shepard J., Retik A.B. (1993). Implantation in vivo and retrieval of artificial structures consisting of rabbit and human urothelium and human bladder muscle. J. Urol..

[6] Oberpenning F., Meng J., Yoo J.J., Atala A. (1999). De novo reconstitution of a functional mammalian urinary bladder by tissue engineering. Nat. Biotechnol..

[7] Lai J.Y., Yoon C.Y., Yoo J.J., Wulf T., Atala A., Kropp B. (2002). Phenotypic and functional characterization of in vivo tissue engineered smooth muscle from normal and pathological bladders. J. Urol..

[8] Nakanishi Y., Chen G., Komuro H., Ushida T., Kaneko S., Tateishi T., Kaneko M. (2003). Tissue-Engineered Urinary Bladder Wall Using PLGA Mesh-Collagen Hybrid Scaffolds: A Comparison Study of Collagen Sponge and Gel as a Scaffold. J. Pediatr. Surg..

[9] Raya-Rivera A., Esquiliano D.R., Yoo J.J., Lopez-Bayghen E., Soker S., Atala A. (2011). Tissue-engineered autologous urethras for patients who need reconstruction: An observational study. Lancet.

[10] Micol L.A., Arenas da Silva L.F., Geutjes P.J., Oosterwijk E., Hubbell J.A., Feitz W.F.J., Frey P. (2012). In-Vivo performance of high-density collagen gel tubes for urethral regeneration in a rabbit model. Biomaterials.

[11] Sayeg K., Freitas-Filho L.G., Waitzberg Â.F.L., Arias V.E.A., Laks M., Egydio F.M., Oliveira A.S. (2013). Integration of collagen matrices into the urethra when implanted as onlay graft. Int. Braz. J. Urol..

[12] Pinnagoda K., Larsson H.M., Vythilingam G., Vardar E., Engelhardt E.M., Thambidorai R.C., Hubbell J.A., Frey P. (2016). Engineered acellular collagen scaffold for endogenous cell guidance, a novel approach in urethral regeneration. Acta Biomater..

[13] Aufderklamm S., Vaegler M., Kelp A., Maurer S., Gustafsson L., Mundhenk J., Busch S., Daum L., Stenzl A., Amend B. (2017). Collagen cell carriers seeded with human urothelial cells for urethral reconstructive surgery: First results in a xenograft minipig model. World J. Urol..

[14] Xie M., Song L., Wang J., Fan S., Zhang Y., Xu Y. (2013). Evaluation of stretched electrospun silk fibroin matrices seeded with urothelial cells for urethra reconstruction. J. Surg. Res..

[15] Algarrahi K., Franck D., Ghezzi C.E., Cristofaro V., Yang X., Sullivan M.P., Chung Y.G., Affas S., Jennings R., Kaplan D.L. (2015). Acellular bi-layer silk fibroin scaffolds support functional tissue regeneration in a rat model of onlay esophagoplasty. Biomaterials.

[16] Yang B., Zhang Y., Zhou L., Sun Z., Zheng J., Chen Y., Dai Y. (2010). Development of a porcine bladder acellular matrix with well-preserved extracellular bioactive factors for tissue engineering. Tissue Eng. Part C Methods.

[17] Kropp B.P., Rippy M.K., Badylak S.F., Adams M.C., Keating M.A., Rink R.C., Thor K.B. (1996). Regenerative urinary bladder augmentation using small intestinal submucosa: Urodynamic and histopathologic assessment in long-term canine bladder augmentations. J. Urol..

[18] Campodonico F., Benelli R., Michelazzi A., Ognio E., Toncini C., Maffezzini M. (2004). Bladder cell culture on small intestinal submucosa as bioscaffold: Experimental study on engineered urothelial grafts. Eur. Urol..

[19] Drewa T. (2007). The Artificial Conduit for Urinary Diversion in Rats: A Preliminary Study. Transplant. Proc..

[20] Wu S., Liu Y., Bharadwaj S., Atala A., Zhang Y. (2011). Human urine-derived stem cells seeded in a modified 3D porous small intestinal submucosa scaffold for urethral tissue engineering. Biomaterials.

[21] Liu Y., Bharadwaj S., Lee S.J., Atala A., Zhang Y. (2009). Optimization of a natural collagen scaffold to aid cell-matrix penetration for urologic tissue engineering. Biomaterials.

[22] Liao W.-B., Song C., Li Y.-W., Yang S.-X., Meng L.-C., Li X.-H. (2013). Tissue-engineered conduit using bladder acellular matrix and bladder epithelial cells for urinary diversion in rabbits. Chin. Med. J..

[23] Pederzoli F., Joice G., Salonia A., Bivalacqua T.J., Sopko N.A. (2019). Regenerative and engineered options for urethroplasty. Nat. Rev. Urol..

[24] Johnson S.C., Smith Z.L., Sack B.S., Steinberg G.D. (2018). Tissue Engineering and Conduit Substitution. Urol. Clin. N. Am..

[25] Casarin M., Fortunato T.M., Imran S., Todesco M., Sandrin D., Borile G., Toniolo I., Marchesan M., Gerosa G., Bagno A. (2022). Porcine Small Intestinal Submucosa (SIS) as a Suitable Scaffold for the Creation of a Tissue-Engineered Urinary Conduit: Decellularization, Biomechanical and Biocompatibility Characterization Using New Approaches. Int. J. Mol. Sci..

[26] Casarin M., Todesco M., Sandrin D., Romanato F., Bagno A., Morlacco A., Moro F.D. (2022). A Novel Hybrid Membrane for Urinary Conduit Substitutes Based on Small Intestinal Submucosa Coupled with Two Synthetic Polymers. J. Funct. Biomater..

[27] European Chemicals Agency (2012). Inclusion of Substances of Very High Concerns in the Candidate List (Decision of the European Chemicals Agency).

[28] Iop L., Bonetti A., Naso F., Rizzo S., Cagnin S., Bianco R., Dal Lin C., Martini P., Poser H., Franci P. (2014). Decellularized allogeneic heart valves demonstrate self-regeneration potential after a long-term preclinical evaluation. PLoS ONE.

[29] Iop L., Paolin A., Aguiari P., Trojan D., Cogliati E., Gerosa G. (2017). Decellularized Cryopreserved Allografts as Off-the-Shelf Allogeneic Alternative for Heart Valve Replacement: In Vitro Assessment before Clinical Translation. J. Cardiovasc. Transl. Res..

[30] Faggioli M., Moro A., Butt S., Todesco M., Sandrin D., Borile G., Bagno A., Fabozzo A., Romanato F., Marchesan M. (2022). A New Decellularization Protocol of Porcine Aortic Valves Using Tergitol to Characterize the Scaffold with the Biocompatibility Profile Using Human Bone Marrow Mesenchymal Stem Cells. Polymers.

[31] Zhao S., Todorov M.I., Cai R., -Maskari R.A., Steinke H., Kemter E., Mai H., Rong Z., Warmer M., Stanic K. (2020). Cellular and Molecular Probing of Intact Human Organs. Cell.

[32] Filippi A., Gintoli M., Filippi A., Sasso E.D., Iop L., Armani A., Gintoli M., Sandri M., Gerosa G., Romanato F. (2018). Multimodal label-free ex vivo imaging using a dual-wavelength microscope with axial chromatic aberration compensation. J. Biomed. Opt..

[33] Borile G., Sandrin D., Filippi A., Anderson K.I., Romanato F. (2021). Label-Free Multiphoton Microscopy: Much more than Fancy Images. Int. J. Mol. Sci..

[34] Schindelin J., Arganda-Carreras I., Frise E., Kaynig V., Longair M., Pietzsch T., Preibisch S., Rueden C., Saalfeld S., Schmid B. (2012). Fiji: An open-source platform for biological-image analysis. Nat. Methods.

[35] Rezakhaniha R., Agianniotis A., Schrauwen J.T.C., Griffa A., Sage D., Bouten C.V.C., Van De Vosse F.N., Unser M., Stergiopulos N. (2012). Experimental investigation of collagen waviness and orientation in the arterial adventitia using confocal laser scanning microscopy. Biomech. Model. Mechanobiol..

[36] Zouhair S., Sasso E.D., Tuladhar S.R., Fidalgo C., Vedovelli L., Filippi A., Borile G., Bagno A., Marchesan M., De Rossi G. (2020). A comprehensive comparison of bovine and porcine decellularized pericardia: New insights for surgical applications. Biomolecules.

[37] Todesco M., Zardin C., Iop L., Palmosi T., Capaldo P., Romanato F., Gerosa G., Bagno A. (2021). Hybrid membranes for the production of blood contacting surfaces: Physicochemical, structural and biomechanical characterization. Biomater. Res..

[38] Bagno A., Aguiari P., Fiorese M., Iop L., Spina M., Gerosa G. (2018). Native Bovine and Porcine Pericardia Respond to Load with Additive Recruitment of Collagen Fibers. Artif. Organs.

[39] Pei M., Zou D., Gao Y., Zhang J., Huang P., Wang J., Huang J., Li Z., Chen Y., Li Z. (2021). The influence of sample geometry and size on porcine aortic material properties from uniaxial tensile tests using custom-designed tissue cutters, clamps and molds. PLoS ONE.

[40] Brauner J.W., Flach C.R., Mendelsohn R. (2005). A quantitative reconstruction of the amide I contour in the IR spectra of globular proteins: From structure to spectrum. J. Am. Chem. Soc..

[41] Todesco M., Imran S.J., Fortunato T.M., Sandrin D., Borile G., Romanato F., Casarin M., Giuggioli G., Conte F., Marchesan M. (2022). A New Detergent for the Effective Decellularization of Bovine and Porcine Pericardia. Biomimetics.

[42] Oldenburg K. LoadSpectra. https://www.mathworks.com/matlabcentral/fileexchange/57904-loadspectra.

[43] Fidalgo C., Iop L., Sciro M., Harder M., Mavrilas D., Korossis S., Bagno A., Palù G., Aguiari P., Gerosa G. (2018). A sterilization method for decellularized xenogeneic cardiovascular scaffolds. Acta Biomater..

[44] (2005). Council of Europe 2.6.1. Sterility. Eur. Pharmacopoeia.

[45] C.S.A. (2009). ISO 10993-5 in vitro cytotoxicity. Int. Organ..

[46] Crapo P.M., Gilbert T.W., Badylak S.F. (2012). An Overview of Tissue and Whole organ decellularization processes. Biomaterials.

[47] Gallagher W. (2009). FTIR Analysis of Protein Structure. Course Man. Chem..

[48] De Campos Vidal B., Mello M.L.S. (2011). Collagen type I amide I band infrared spectroscopy. Micron.

[49] Lazarev Y.A., Grishkovsky B.A., Khromova T.B. (1985). Amide I band of IR spectrum and structure of collagen and related polypeptides. Biopolymers.

[50] Shabsigh A., Korets R., Vora K.C., Brooks C.M., Cronin A.M., Savage C., Raj G., Bochner B.H., Dalbagni G., Herr H.W. (2009). Defining Early Morbidity of Radical Cystectomy for Patients with Bladder Cancer Using a Standardized Reporting Methodology. Eur. Urol..

[51] Pruthi R.S., Nielsen M., Smith A., Nix J., Schultz H., Wallen E.M. (2010). Fast Track Program in Patients Undergoing Radical Cystectomy: Results in 362 Consecutive Patients. J. Am. Coll. Surg..

[52] Ramirez J.A., McIntosh A.G., Strehlow R., Lawrence V.A., Parekh D.J., Svatek R.S. (2013). Definition, incidence, risk factors, and prevention of paralytic ileus following radical cystectomy: A systematic review. Eur. Urol..

[53] Faba O.R., Moreno R.P., Malca L., Martínez A.P., Nervo N., Breda A., Esquinas C., Palou J. (2018). Postoperative management of radical cystectomy. Review of the evidence on the prevention and treatment of urological complications. Actas Urológicas Españolas.

[54] Gilbert T.W., Sellaro T.L., Badylak S.F. (2006). Decellularization of tissues and organs. Biomaterials.

[55] Casarin M., Morlacco A., Dal Moro F. (2021). Bladder Substitution: The Role of Tissue Engineering and Biomaterials. Processes.

[56] Casarin M., Morlacco A., Moro F.D. (2022). Tissue Engineering and Regenerative Medicine in Pediatric Urology: Urethral and Urinary Bladder Reconstruction. Int. J. Mol. Sci..

[57] Rosenberg M.L., Dahlen G.A. (1953). Autogenous vein grafts and venous valves in ureteral surgery; an experimental study. J. Urol..

[58] Schein C.J., Sanders A.R., Hurwitt E.S. (1955). The fate of fresh autogenous arterial grafts embedded in submucosal intestinal tunnels as applied to the bridging of ureteral defects. Ann. Surg..

[59] Sewell W.H. (1955). Failure of freeze-dried homologous arteries used as ureteral grafts. J. Urol..

[60] Albert P.S., Vitolo R.V., Friedenberg R., Davis J.E. (1976). Bovine carotid heterograft for segmental ureteral substitution. Am. J. Surg..

[61] Kloskowski T., Jundziłł A., Kowalczyk T., Nowacki M., Bodnar M., Marszałek A., Pokrywczyńska M., Frontczak-Baniewicz M., Kowalewski T.A., Chłosta P. (2014). Ureter regeneration-The proper scaffold has to be defined. PLoS ONE.

[62] Kloskowski T., PokrywczyŃska M., Drewa T. (2015). Artificial urinary conduit construction using tissue engineering methods. Cent. Eur. J. Urol..

[63] Guler S., Aydin H.M., Lü L.X., Yang Y. (2018). Improvement of Decellularization Efficiency of Porcine Aorta Using Dimethyl Sulfoxide as a Penetration Enhancer. Artif. Organs.

[64] Walawalkar S., Almelkar S. (2019). Fabrication of aortic bioprosthesis by decellularization, fibrin glue coating and re-endothelization: A cell scaffold approach. Prog. Biomater..

[65] Kanda H., Ando D., Hoshino R., Yamamoto T., Wahyudiono, Suzuki S., Shinohara S., Goto M. (2021). Surfactant-Free Decellularization of Porcine Aortic Tissue by Subcritical Dimethyl Ether. ACS Omega.

[66] Thiene G., Basso C., Della Barbera M. (2021). Pathology of the aorta and aorta as homograft. J. Cardiovasc. Dev. Dis..

[67] Guler S., Aslan B., Hosseinian P., Aydin H.M. (2017). Supercritical Carbon Dioxide-Assisted Decellularization of Aorta and Cornea. Tissue Eng.—Part C Methods.

[68] Lotze M.T., Deisseroth A., Rubartelli A. (2007). Damage associated molecular pattern molecules. Clin. Immunol..

[69] Gilpin A., Yang Y. (2017). Decellularization Strategies for Regenerative Medicine: From Processing Techniques to Applications. BioMed Res. Int..

[70] Chow M.J., Turcotte R., Lin C.P., Zhang Y. (2014). Arterial extracellular matrix: A mechanobiological study of the contributions and interactions of elastin and collagen. Biophys. J..

[71] Dingemans K.P., Teeling P., Lagendijk J.H., Becker A.E. (2000). Extracellular matrix of the human aortic media: An ultrastructural histochemical and immunohistochemical study of the adult aortic media. Anat. Rec..

[72] Tsamis A., Krawiec J.T., Vorp D.A. (2013). Elastin and collagen fibre microstructure of the human aorta in ageing and disease: A review. J. R. Soc. Interface.

[73] Deplano V., Boufi M., Boiron O., Guivier-Curien C., Alimi Y., Bertrand E. (2016). Biaxial tensile tests of the porcine ascending aorta. J. Biomech..

[74] Zeinali-Davarani S., Wang Y., Chow M.J., Turcotte R., Zhang Y. (2015). Contribution of collagen fiber undulation to regional biomechanical properties along porcine thoracic aorta. J. Biomech. Eng..

[75] Tanaka T., Fung Y.C. (1974). Elastic and inelastic properties of the canine aorta and their variation along the aortic tree. Fed. Proc..

[76] Purslow P.P. (1983). Positional variations in fracture toughness, stiffness and strength of descending thoracic pig aorta. J. Biomech..

[77] Ma P.X. (2004). Scaffolds for tissue fabrication. Mater. Today.

[78] Łopianiak I., Butruk-Raszeja B.A. (2020). Evaluation of sterilization/disinfection methods of fibrous polyurethane scaffolds designed for tissue engineering applications. Int. J. Mol. Sci..

[79] Dai Z., Ronholm J., Tian Y., Sethi B., Cao X. (2016). Sterilization techniques for biodegradable scaffolds in tissue engineering applications. J. Tissue Eng..

[80] Hussein K.H., Park K.M., Kang K.S., Woo H.M. (2016). Biocompatibility evaluation of tissue-engineered decellularized scaffolds for biomedical application. Mater. Sci. Eng. C.

[81] Wang C., Li Y., Yang M., Zou Y., Liu H., Liang Z., Yin Y., Niu G., Yan Z., Zhang B. (2018). Efficient Differentiation of Bone Marrow Mesenchymal Stem Cells into Endothelial Cells in vitro. Eur. J. Vasc. Endovasc. Surg..

[82] Gu W., Hong X., Le Bras A., Nowak W.N., Bhaloo S.I., Deng J., Xie Y., Hu Y., Ruan X.Z., Xu Q. (2018). Smooth muscle cells differentiated from mesenchymal stem cells are regulated by microRNAs and suitable for vascular tissue grafts. J. Biol. Chem..

[83] Oswald J., Boxberger S., Joergensen B., Bornhaeuser M., Ehninger G., Werner C. (2004). Mesenchymal Stem Cells (MSC) can be differentiated into endothelial cells in vitro. Stem Cells..

