# Supplementary material for: Preliminary In Vitro Assessment of Decellularized Porcine Descending Aorta for Clinical Purposes
Source: J Funct Biomater. 2023 Mar 2;14(3):141. doi: 10.3390/jfb14030141 (PMC10058365; doi:10.3390/jfb14030141)
Supplement: Supplementary file 1 [file jfb-14-00141-s001.zip › jfb-2227908-supplementary.pdf]

Journal of
Functional
Biomaterials

Supplementary Materials

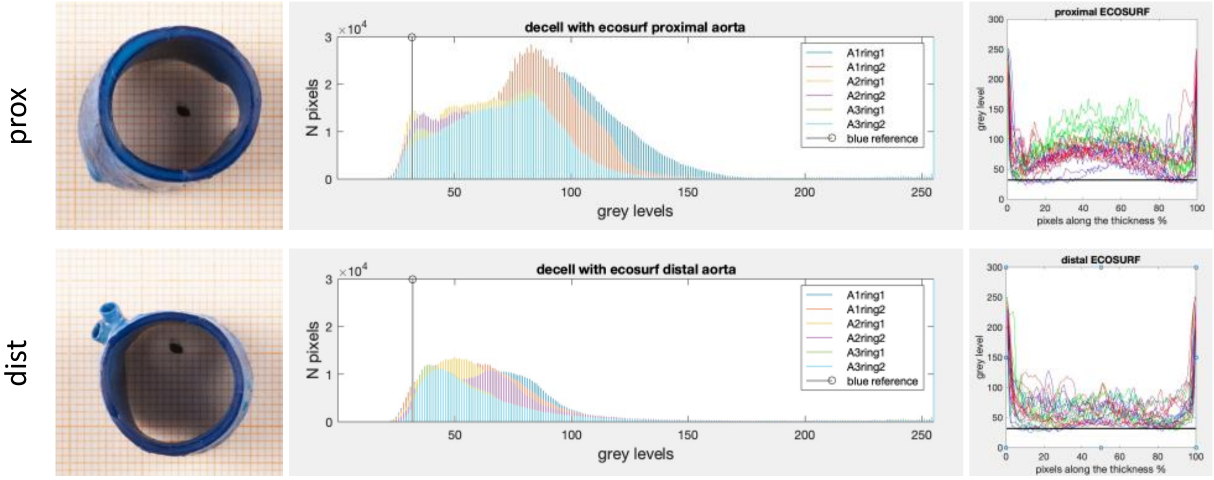

Figure S1: Penetration assessment of methylene blue dye in Ecosurf–treated samples at the end of the third step of the decellularization procedure in proximal and distal regions of descending aorta. Cross–sectional pictures were segmented and analyzed in order to obtain the histograms of occurrence of each grey level and then the graphs of grey levels along the thickness (%) of samples. The vertical black lines in histograms and horizontal black lines in graphs represent the reference grey level of methylene blue dye. Greyscale: 0=black, 255=white.

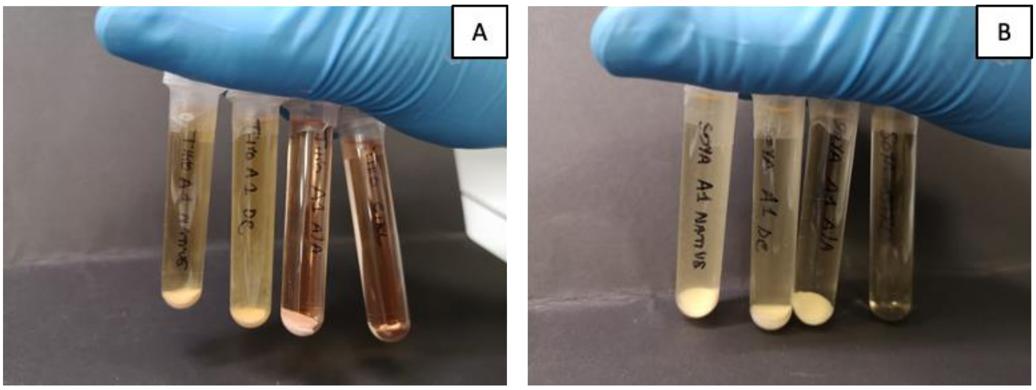

Figure S2: Sterility assessment. Representative samples of turbidity tests of native, only decellularized and decellularized + sterilized samples are reported in comparison with only turbidity media (blank) both for Thioglycollate medium (**A**) and Soya Broth medium (**B**) at day 14. Positiveness to turbidity was found in both media in the case of native and only decellularized samples, while sterilized samples gave no evidence of turbidity.
